# Dietary intake and serum concentrations of vitamin A and vitamin E and pre-eclampsia risk in Chinese pregnant women: A matched case-control study

**DOI:** 10.3389/fnut.2023.1049055

**Published:** 2023-03-30

**Authors:** Yanhua Liu, Shunping Ma, Xuemin Huang, Yacong Bo, Wenjun Fu, Yuan Cao, Dandan Duan, Weifeng Dou, Fangfang Zeng, Xinyi Wang, Meiyuan Gong, Xueyang Zhang, Quanjun Lyu, Xianlan Zhao

**Affiliations:** ^1^Department of Nutrition, The First Affiliated Hospital of Zhengzhou University, Zhengzhou, China; ^2^Department of Nutrition and Food Hygiene, College of Public Health, Zhengzhou University, Zhengzhou, China; ^3^Department of Obstetrics, The First Affiliated Hospital of Zhengzhou University, Zhengzhou, China; ^4^The Third Affiliated Hospital of Zhengzhou University, Zhengzhou, China; ^5^Department of Clinical Nutrition, Luoyang New Area People's Hospital, Luoyang, China; ^6^Department of Epidemiology, School of Medicine, Jinan University, Guangzhou, China; ^7^School of Pharmaceutical Sciences, Zhengzhou University, Zhengzhou, China

**Keywords:** vitamin A, vitamin E, pre-eclampsia, Chinese, a case-control study

## Abstract

**Background:**

Many studies have suggested that the serum concentrations of vitamin A (VA) and vitamin E (VE) influence preeclampsia (PE) risk in pregnant women. However, few studies have assessed whether dietary intake and serum concentrations of VA and VE are correlated with PE risk.

**Methods:**

A 1:1 matched case-control study was conducted to explore the association between the dietary intake and serum concentrations of VA and VE and the risk of PE in pregnant Chinese women. A total of 440 pregnant women with PE and 440 control pregnant women were included in the study. Dietary information was obtained using a 78-item semi-quantitative food frequency questionnaire. Serum concentrations of VA and VE were measured by liquid chromatography-tandem mass spectrometry.

**Results:**

Compared with the lowest quartile, the multivariate-adjusted odds ratios [95% confidence interval (CI)] of the highest quartiles were 0.62 (95% CI: 0.40-0.96, *P* trend = 0.02) for VA, 0.51 (95% CI: 0.33–0.80, *P* trend =0.002) for β-carotene, and 0.70 (95% CI: 0.45–1.08, *P* trend = 0.029) for retinol. Additionally, for serum VA and VE concentrations, the multivariate-adjusted odds ratios (95% CI) were 2.75 (95% CI: 1.24–6.13, *P* trend = 0.002) and 11.97 (95% CI: 4.01–35.77, *P* trend < 0.001), respectively. No significant association was seen between VE intake and PE risk.

**Conclusions:**

Dietary VA intake was negatively correlated with PE risk, and serum VA and VE concentrations were positively correlated with PE risk among pregnant Chinese women.

## 1. Introduction

Pre-eclampsia (PE) is a pregnancy complication that occurs after 20 weeks of gestation and is characterized by hypertension, proteinuria, and multiple organ dysfunction (1). It has been reported that approximately 2–8% of first pregnancies are diagnosed with PE, and is the most important cause of perinatal maternal and infant mortality (2). Meanwhile, PE is a major risk factor for poor maternal and child health and substantially increases the risk of miscarriage, acute renal failure, and low birth weight stillbirth (3). Currently, termination of the pregnancy is the only treatment available for PE. Therefore, finding effective ways to prevent PE is crucial.

Vitamin A (VA) and vitamin E (VE) are fat-soluble vitamins (4), with retinol and carotene being the most common forms of VA (5) and alpha- and gamma-tocopherol being the predominant forms of VE in humans (6). Retinol is mainly derived from animal foods, while carotene is derived from plants (7). VE is mainly derived from vegetable oils, unprocessed grains, nuts, fruits, and vegetables (8). Recent studies have shown that VA and VE play important roles in antioxidation, inflammation, vision, immune health, and anti-apoptotic cellular processes (9–11). Antioxidative stress and anti-inflammatory responses are associated with PE in pregnant women (12), suggesting an association between VA, VE, and PE. Several epidemiological studies have suggested that VA and VE concentrations in maternal circulation, umbilical cord blood, and the blood serum of women with PE are lower than those seen in women with normal pregnancies (13–17). Some studies have shown that VA and VE supplementation during pregnancy can protect against PE (18–21). Our previous studies have found that dietary carotenoid intake is associated with PE risk (22). In contrast, some studies have suggested that there is no association between the risk of gestational hypertensive disorders and dietary VE intake (23). However, such inconsistent results are possibly due to differences in the study populations, methods of assessing dietary nutrients, covariate adjustments, and sample sizes. Moreover, the dietary patterns differ among these studies (24). A recently conducted review showed that dietary VE or retinoic acid can protect women with low-risk pregnancies against developing PE (25). A previous retrospective study of 73,317 participants and 1,671 cases of PE found a positive correlation between a reduced serum VE concentration (< 7.3 mg/L) and PE (26). However, that study only included cases of PE reported during the first trimester. The current case–control study was conducted to explore the association between dietary and serum concentrations of VA and VE with PE risk in pregnant Chinese women. We hypothesized that a dose–response relationship exists between dietary and serum VA and VE and PE risk.

## 2. Methods

### 2.1. Study participants

This 1:1 matched case–control study was performed in the First Affiliated Hospital of Zhengzhou University, China, from March 2016 to June 2019. The study design was described previously (27). Cases were defined as women diagnosed with PE based on China's “Diagnosis and treatment guideline of hypertensive disorders in pregnancy (2015)” (28). In this guideline, PE is defined as systolic blood pressure (SBP) of ≥140 mmHg or diastolic blood pressure (DBP) of ≥90 mmHg after 20 weeks of gestation, accompanied by any of the following characteristics: (1) urinary protein ≥0.3 g/24 h, or a urinary protein/creatinine ratio ≥0.3, or random urinary protein ≥ (+) (the test method used when urinary protein cannot be quantified); (2) non-albuminuria but with damage to organs or systems such as the heart, lung, liver, kidney, and other important organs, or abnormal changes in the blood system, digestive system, nervous system, and placental–fetal involvement. Pregnant women from the same hospital without hypertension or proteinuria were enrolled as controls and matched with the case group based on age (±3 years), gestational weeks (±1 week), and gestational diabetes mellitus (GDM) status. The exclusion criteria for participants were as follows: (1) refusal to participate in the study; (2) heart disease, malignant tumor(s), hyperthyroidism, an immune system disease, chronic renal insufficiency, or other chronic diseases; and (3) mental or cognitive disorders such as schizophrenia or depression.

This study was approved by the Ethics Committee of Scientific Research and Clinical Trials of the First Affiliated Hospital of Zhengzhou University (No. Scientific research 2016-LW-34). All participants provided written informed consent before epidemiological data and biological specimens were collected. All procedures were performed according to the Declaration of Helsinki guidelines and regulations.

### 2.2. Calculation of sample size

The sample size of this 1:1-matched case–control study was calculated based on the OR estimated from previous studies (OR = 0.45) (29). A sample size of 134 was calculated based on the aforementioned assumptions.

With 80% statistical power and 0.05 two-sided significance level, the sample size of each group was estimated to be 134. This study included 440 cases and 440 controls, thereby meeting the sample size requirements.

### 2.3. Data collection

A structured questionnaire was used to collect information about sociodemographic characteristics (age, weeks of gestation, marital status, educational level, and household income) and dietary intake. The participants' height (m), weight (kg), and blood pressure were measured using digital scales, and the body mass index (BMI, kg/m^2^) was calculated. Gestational age was calculated from the 1st day of the last menstrual period. Passive smokers were defined as participants who had been exposed to exhaled smoke for at least 5 min/d over the past few years.

### 2.4. Assessment of dietary VA and VE intake

The dietary intake of VA and VE during the 3 months prior to giving birth was assessed using the semi-quantitative Food Frequency Questionnaire (FFQ) (30), which includes 78 foods commonly consumed by Chinese people. The intake frequency (0 = never; 1 = per month; 2 = per week; and 3 = per day) and the amount consumed of each food were recorded. The consumed nutrients (μg or mg/day) and energy (kcal/day) were calculated based on the Chinese Food Composition Tables 2004 (31), including the nutrients and energy contained within each food item.

The correlation coefficients between the FFQ and six 3-day dietary records were 0.32 for VA and 0.25 for VE (30, 32).

### 2.5. Laboratory analysis of serum VA and VE concentrations

Blood samples were collected on the day of delivery, and the blood collection criteria were the same. The samples were centrifuged at 2,500 rpm at 4°C for 10 min to separate the sera, and serum samples were stored at −80°C. Serum concentrations of VA and VE were determined by liquid chromatography with tandem mass spectrometry (14). In brief, the serum samples (200 μl) were mixed with an internal standard solution (400 μl) and vortexed for 60 s, and vitamins were then extracted using hexane and centrifugation (12,000 rpm, 5 min). The supernatants were decanted and evaporated under a stream of nitrogen gas until dry. The resulting extract was dissolved in ethanol (100 μl) and analyzed by liquid chromatography (Shimadzu, Kyoto, Japan) with tandem mass spectrometry (AB Sciex, Framingham, MA, United States) to determine the serum concentrations of VA and VE. For chromatographic separation, 0.1% formic acid solution and 0.1% methanol solution of formic acid were used as mobile phases A and B, respectively. Mass spectrometry analyses of VA and VE were performed using positive electrospray ionization and multiple reaction monitoring modes. All procedures were performed by the same technician who was blinded to the participants' case–control status.

### 2.6. Statistical analysis

Unpaired *t*-tests or Wilcoxon signed-rank tests were used to test differences in quantitative variables, and unpaired chi-squared tests were used to identify differences in qualitative variables between cases and controls. The dietary intake data were adjusted for total energy intake using the residual method (33).

According to the distribution among the controls, the dietary VA and VE intake and serum VA and VE concentrations were divided into quartiles (Q1–Q4). ORs and 95% confidence intervals (CIs) for the associations of dietary VA and VE intake and serum VA and VE concentrations with PE risk were estimated using multivariate conditional logistic regression models. Tests for trends were performed by using the median of each quartile as a continuous variable in the regression models.

Potential confounders were adjusted for in the multivariate models, including age, gestational age, pre-pregnancy BMI, family history of hypertension (yes or no), education level (primary school or less, secondary/high school, college/university, or above), parity (0 births, 1 birth, ≥2 births), physical activity, and daily energy intake. A sensitivity analysis of the relationship between dietary VA and VE intake and PE risk was performed by excluding participants with GDM. Potential non-linear associations of dietary and serum VA and VE concentrations with PE risk were examined using restricted cubic spline (RCS) analysis. The 20th, 50th, and 80th percentiles were retained as knots. The RCS was calculated using R 4.0.3. All other analyses were performed using SPSS 25.0 (SPSS Inc., Chicago, IL, United States). A two-tailed *P-*value of < 0.05 was considered statistically significant. The missing values in our study were ignored as they were < 10%.

## 3. Results

### 3.1. Baseline characteristics

The demographic characteristics and PE-related factors of 440 cases and controls are described in Table 1. There were no significant differences identified between PE cases and controls in terms of age (cases vs. controls: 30.9 ± 5.03 years vs. 31.0 ± 4.85 years, *P* = 0.114), gestational week (cases vs. controls: 34.2 ± 2.90 weeks vs. 34.2 ± 2.67 weeks, *P* = 0.066), energy-adjusted dietary VE intake (cases vs. controls: 30.39 mg/d vs. 30.90 mg/d, *P* = 0.310), GDM (cases vs. controls: 59 (13.0) vs. 59 (13.0), *P* = 1.000), polycystic ovarian syndrome (cases vs. controls: 10 (2.3) vs. 6 (1.4), *P* = 0.454), income (*P* = 0.405), physical activity (*P* = 0.241), or multivitamin supplement user (*P* = 0.177). Compared with the control group, PE patients had a higher frequency of a family history of hypertension and greater pre-pregnancy BMI (*P* < 0.001), and a lower educational level (*P* = 0.014) and daily energy intake (*P* = 0.001). The median energy-adjusted dietary VA intake (*P* < 0.001), β-carotene intake (*P* < 0.001), and retinol intake (*P* = 0.008) during the last 3 months prior to delivery were higher in the controls than in the cases.

**Table 1:** Sociodemographic and lifestyle characteristics and selected PE risk factors of the study population (*n* = 440 pairs).

	**Cases (*n* = 440)**	**Controls (*n* = 440)**	***P^*a*^***
Age (years)^b^	30.9 ± 5.03	31.0 ± 4.85	0.114
Gestational age (weeks)^b^	34.2 ± 2.90	34.2 ± 2.67	0.066
Pre-pregnancy BMI (kg/m^2^)^b^	23.7 ± 3.89	22.4 ± 3.35	< 0.001
Gestational diabetes mellitus^c^	59 (13.0)	59 (13.0)	1.000
Polycystic ovarian syndrome^c^	10 (2.3)	6 (1.4)	0.454
Family history of hypertension	167 (38.0)	83 (18.9)	< 0.001
**Education level** ^c^			0.014
Junior high school or below	207 (47.0)	164 (37.4)	
Senior high school	75 (17.0)	83 (18.9)	
College or above	158 (35.9)	192 (43.7)	
**Income (Yuan/month)** ^c^			0.405
≤ 2,000	61 (13.9)	46 (10.5)	
2,001–4,000	216 (49.1)	211 (48.0)	
4,001–6,000	78 (17.7)	82 (18.6)	
>6,000	59 (13.4)	81 (18.4)	
**Passive smoker** ^c^	67 (15.2)	58 (13.2)	0.488
**Parity**			0.001
0 births	185 (42.0)	135 (30.7)	
1 birth	180 (40.9)	211 (48.0)	
≥2 births	73 (16.6)	93 (21.1)	
Physical activity (MET-h/day)^b^	27.0 ± 3.96	26.6 ± 4.48	0.241
Daily energy intake (kcal/day)^b^	1,850 ± 504	1,962 ± 521	0.001
Multivitamin supplement user^c^	138 (31.4)	158 (35.9)	0.177
Dietary VA intake (μg RE/day)^d^	736 (568, 952)	820 (631, 1,053)	< 0.001
Dietary β-carotene intake (μg/day)^d^	6,265 (4,756, 8,287)	7,097 (5,366, 9,712)	< 0.001
Dietary retinol intake (μg/day)^d^	173 (107, 263)	204 (131, 298)	0.008
Dietary VE intake (mg/day)^d^	30.4 (24.6, 36.1)	30.9 (24.8, 36.8)	0.310

MET, metabolic equivalent; BMI, body mass index; SD, standard deviation; VE, vitamin E; VA, vitamin A.

^a^Continuous variables were evaluated using paired *t*-tests or Wilcoxon rank-sum tests. Categorical variables were evaluated using paired chi-squared tests.

^b^Data are presented as the mean ± standard deviation.

^c^Data are presented as the number (%).

^d^Data are presented as the M (P25, P75).

### 3.2. Serum concentrations

The serum concentrations of VA and VE among participants are shown in Table 2. Compared with the control group, PE patients had greater serum concentrations of VA (cases vs. controls: 318.11 ± 146.34 ng/ml vs. 268.7 ± 125.0 ng/ml, *P* = 0.003) and VE (cases vs. controls: 15,101 ± 4,664 ng/ml vs. 12,563 ± 4,738 ng/ml, *P* = 0.003) (Table 2). No significant differences in age (*P* = 0.518), gestational age (*P* = 0.058), or pre-pregnancy BMI (*P* = 0.049) were seen in participants whose blood samples were collected (Table 2).

**Table 2:** Sociodemographic and lifestyle characteristics and selected PE risk factors of participants whose blood samples were collected (*n* = 150 pairs).

	**Cases (*n* = 150)**	**Controls (*n* = 150)**	***P* ^a^**
Age (years)^b^	31.4 ± 4.75	31.3 ± 4.54	0.518
Gestational age (weeks)^b^	34.1 ± 2.77	34.2 ± 2.69	0.058
Pre-pregnancy BMI (kg/m^2^)^b^	23.5 ± 3.90	22.7 ± 3.48	0.049
Serum concentration of Vitamin A (ng/ml)^b^	318± 146	269 ± 125	0.003
Serum concentration of Vitamin E (ng/ml)^b^	15,101 ± 4,664	12,563 ± 4,738	< 0.001

BMI, body mass index.

^a^Continuous variables were evaluated using paired *t*-tests or Wilcoxon rank-sum tests. Categorical variables were evaluated using paired chi-squared tests.

^b^Data are presented as the mean ± standard deviation.

### 3.3. Dietary VA and VE intake and PE risk

Dietary VA intake was negatively correlated with PE risk (Table 3). After adjusting for possible confounders, the OR for PE in the highest quartile relative to the lowest quartile was 0.62 (95% CI: 0.40–0.96, *P* trend = 0.020) for dietary VA intake, 0.51 (95% CI: 0.33–0.80, *P* trend = 0.002) for dietary β-carotene intake, and 0.70 (95% CI: 0.45–1.08, *P* trend = 0.029) for dietary retinol intake (Table 3). Sensitivity analysis results are shown in Supplementary Table 1. After excluding 58 participant case–control pairs with GDM, no substantial changes were observed in the relationship between dietary VA intake and PE risk. No significant associations were seen between dietary VE intake and PE risk, both with and without adjustment for covariates (Table 3).

**Table 3:** Odds ratios and 95% confidence intervals for PE risk according to dietary VA and VE intake quartiles (*n* = 440 pairs).

	**Q1**	**Q2**	**Q3**	**Q4**	***P* trend^b^**
**Dietary VA intake**					
Median (μg RE/day)^a^	523	742	921	1,285	-
Cases/controls	157/110	114/110	86/110	83/110	-
Crude OR	1	0.71 (0.49–1.02)	0.58 (0.40–0.83)	0.54 (0.37–0.79)	0.001
Adjusted OR^c^	1	0.83 (0.54–1.28)	0.68 (0.44–1.03)	0.62 (0.40–0.96)	0.020
**Dietary** β**-carotene intake**					
Median (μg/day)^a^	4,231	6,284	8,071	11,581	-
Cases/controls	151/110	116/110	101/110	72/110	-
Crude OR	1	0.76 (0.53–1.09)	0.67 (0.46–0.98)	0.48 (0.32–0.70)	< 0.001
Adjusted OR^c^	1	0.86 (0.56–1.32)	0.63 (0.40–0.98)	0.51 (0.33–0.80)	0.002
**Dietary retinol intake**					
Median (μg/day)^a^	77.5	168	245	386	-
Cases/controls	145/110	127/110	81/110	87/110	-
Crude OR	1	0.86 (0.60–1.25)	0.55 (0.37–0.81)	0.60 (0.41–0.88)	0.003
Adjusted OR ^c^	1	1.09 (0.70–1.69)	0.59 (0.38–0.93)	0.70 (0.45–1.08)	0.029
**Dietary VE intake**					
Median (mg/day)^a^	21.3	28.3	33.5	41.7	-
Cases/controls	113/110	116/110	112/110	99/110	-
Crude OR	1	1.02 (0.72–1.47)	0.99 (0.69–1.41)	0.88 (0.61–1.27)	0.481
Adjusted OR^c^	1	0.88 (0.58–1.33)	0.95 (0.63–1.43)	0.74 (0.48–1.13)	0.208

VE, vitamin E; VA, vitamin A; OR, odds ratio; CI, confidence interval.

^a^Median intake in controls, which was subsequently adjusted for daily energy intake.

^b^Determined by entering the median intake for each quartile as a continuous variable in the regression models.

^c^OR adjusted for age, gestational age, pre-pregnancy BMI, family history of hypertension, education level, parity, physical activity, and daily energy intake.

**Figure 1:**
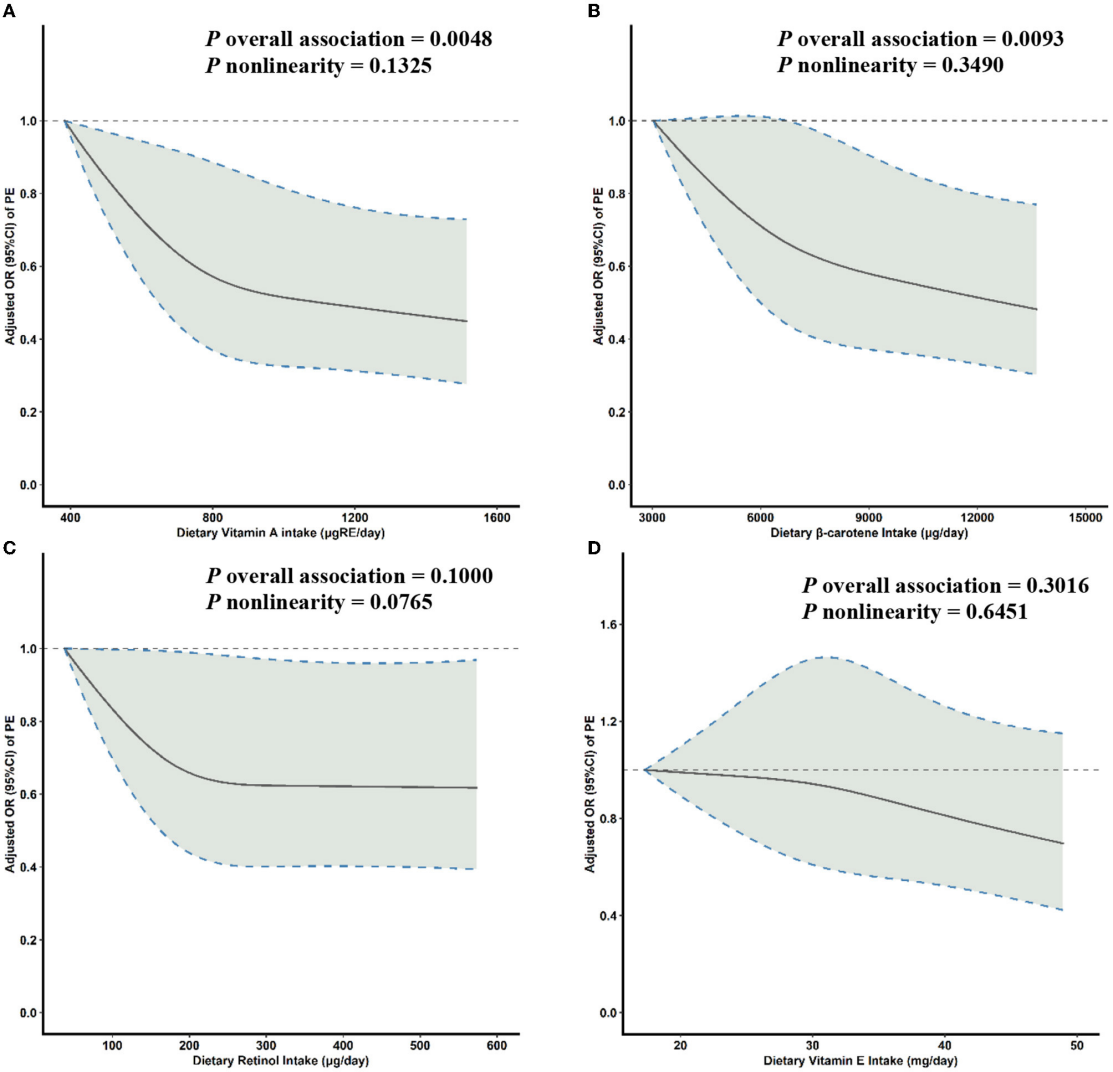
Multivariable-adjusted ORs (solid lines) and 95% CIs (dashed lines) for PE risk according to dietary **(A)** VA, **(B)** β-carotene, **(C)** retinol, and **(D)** VE intake. The model was adjusted for age, gestational age, pre-pregnancy BMI, family history of hypertension, education level, parity, physical activity, and daily energy intake. OR, odds ratio; CI, confidence interval; PE, pre-eclampsia; VA, vitamin A; VE, vitamin E; BMI, body mass index.

Multivariable-adjusted RCS analyses suggested a reverse J-shaped relationship between both dietary VA intake and β-carotene intake and PE risk (Figures 1A, B). With increasing levels of daily intake, the risk of PE initially decreased sharply and then plateaued after the inflection points of 800 μg of retinol equivalents (RE)/day for VA (*P* overall association = 0.0048, *P* non-linearity = 0.1325) and 8,050 μg/day for β-carotene (*P* overall association = 0.0093, *P* non-linearity = 0.3490) (Figure 1). No significant associations were seen between retinol or VE intake and PE risk (Figures 1C, D).

### 3.4. Serum concentrations of VA and VE and PE risk

Table 4 shows the ORs and 95% CIs of PE risk stratified by serum VA and VE concentration quartiles. Significant positive dose-dependent associations were seen for serum VA and VE concentrations in both univariate and multivariate models. Compared with the lowest quartiles, the adjusted ORs for PE of the highest quartile were 2.75 (95% CI: 1.24–6.13, *P* trend = 0.002) and 11.97 (95% CI: 4.01–35.8, *P* trend < 0.001) for serum VA and VE concentrations, respectively.

**Table 4:** Odds ratios and 95% confidence intervals for PE according to serum VA and VE concentration quartiles among participants whose blood samples were collected (*n* = 150 pairs).

	**Q1**	**Q2**	**Q3**	**Q4**	***P* trend^b^**
**Serum concentrations of vitamin A**					
Median (ng/ml)^a^	138	221	286	413	-
Cases/controls	25/37	27/38	36/38	62/37	-
Crude OR	1	1.09 (0.55–2.19)	1.36 (0.70–2.65)	2.21 (1.17–4.16)	0.004
Adjusted OR^c^	1	0.91 (0.40–2.10)	1.66 (0.72–3.79)	2.75 (1.24–6.13)	0.002
**Serum concentrations of vitamin E**					
Median (ng/ml)^a^	8,560	10,650	12,450	17,800	-
Cases/controls	8/37	27/38	44/38	71/37	-
Crude OR	1	3.36 (1.24–9.11)	5.04 (1.99–12.8)	8.34 (3.30–21.2)	< 0.001
Adjusted OR^c^	1	5.84 (1.77–19.3)	7.15 (2.42–21.1)	12.0 (4.01–35.8)	< 0.001

OR, odds ratio; CI, confidence interval.

^a^Median intake in controls, which was subsequently adjusted for daily energy intake.

^b^Determined by entering the median intake for each quartile as a continuous variable in the regression models.

^c^OR adjusted for age, gestational age, pre-pregnancy BMI, family history of hypertension, education level, parity, physical activity, and daily energy intake.

**Figure 2:**
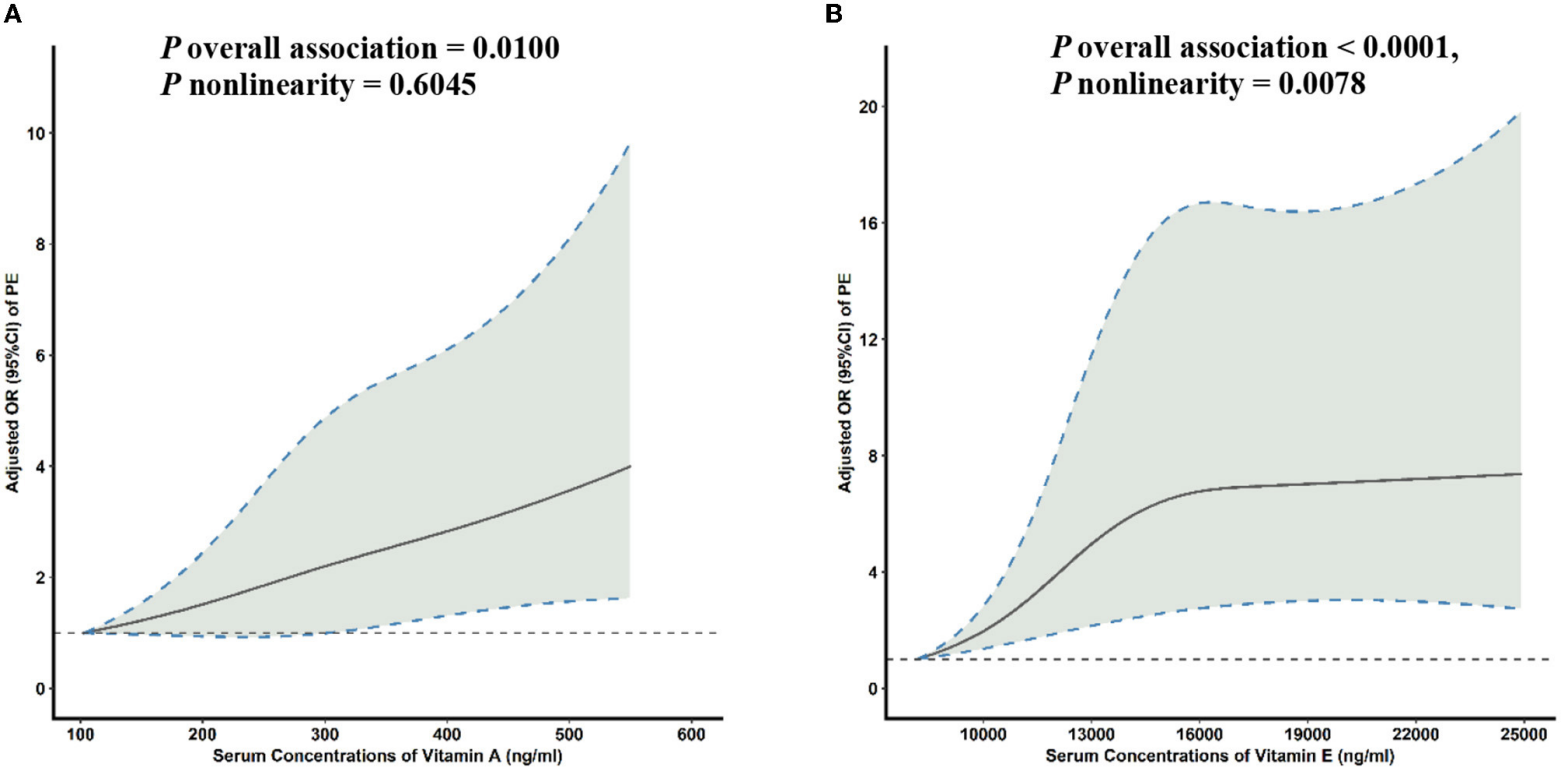
Multivariable-adjusted ORs (solid lines) and 95% CIs (dashed lines) for PE risk according to serum concentrations of **(A)** VA and **(B)** VE. The model was adjusted for age, gestational age, pre-pregnancy BMI, family history of hypertension, education level, parity, physical activity, and daily energy intake. OR, odds ratio; CI, confidence interval; PE, pre-eclampsia; VA, vitamin A; VE, vitamin E; BMI, body mass index.

Multivariable-adjusted RCS analyses revealed a linear association between the serum VA concentration and PE risk (Figure 2A). With increasing concentrations of serum VA, PE risk increased sharply. In addition, significant non-linear associations were seen between the serum VE concentration and PE risk (Figure 2B). With increasing concentrations of serum VE, PE risk initially increased sharply and then plateaued after inflection points of 15,000 ng/ml (*P* overall association = 0.0001, *P* non-linearity = 0.0078).

## 4. Discussion

This 1:1 matched case–control study found that dietary VA intake was negatively correlated with PE risk, and serum VA and VE concentrations were positively correlated with PE risk among pregnant Chinese women. No significant association was seen between VE intake and PE risk. Our findings have important public health implications for the prevention of PE in this population.

Evidence regarding the relationship between dietary VA intake and PE risk is limited. Previous studies have reported that pregnant women diagnosed with PE had a significantly lower intake of β-carotene than those without PE (20). A randomized trial in Nepal found that supplementation with either VA (RR = 0.60; 95% CI: 0.37–0.97) or β-carotene (RR = 0.51; 95% CI: 0.30–0.86) decreased pregnancy-associated mortality rates (34). A prospective cohort study in China including 12,245 participants found that those with higher dietary VA intake (≥227 μg RE/day) had a lower risk of new-onset hypertension (adjusted HR: 0.73; 95% CI: 0.63–0.78) (35). Our results are consistent with previous studies, with the RCS curves suggesting reverse J-shaped associations between the dietary intake of VA and β-carotene and PE risk. Thus, VA intake may protect against PE in pregnant Chinese women.

The association between VE intake and PE risk has been explored by several different studies, but the results have been inconsistent. A case–control study conducted in China including 10,228 gestational women reported no association between the risk of gestational hypertensive disorders and dietary VE intake (23). Similarly, a different case–control study found that dietary VE intake was not significantly different between the pre-eclamptic and control groups (11.74 ± 9.39 vs. 11.34 ± 7.51 mg/24 h, *P* = 0.73) (36). Moreover, a randomized clinical trial reported that VE supplementation during pregnancy did not affect PE risk (37). These findings are supported by our study. We found no evidence supporting a significant association between dietary VE intake and PE risk (*P* = 0.481). However, a study conducted in Australia showed that lower VE intake was associated with an increased risk of hypertensive disorders (RR =1.75, 95% CI: 1.11–2.75, *P* = 0.02) (38). Furthermore, a case–control study suggested that VE supplementation during pregnancy reduced PE incidence (supplementary vs. control group: 7% vs. 13%, *P* < 0.05) (39). The inconsistencies between these studies and our findings may be due to differences in the dietary VE intake concentrations, lifestyles, and dietary patterns, and the limited sample sizes.

Previous studies have assessed the associations between serum VA and VE concentrations and PE risk. Some studies have reported that the plasma, maternal blood, umbilical cord blood, and tissues of pregnant women with PE have lower VA and VE concentrations than those of healthy pregnant women (all *P* < 0.05) (40–43). Our findings disagree with these previous epidemiological studies, as we observed elevated serum VA (case vs. control: 318 ± 146 vs. 269 ± 125, *P* = 0.003) and VE (case vs. control: 15,101 ± 4,664 vs. 12,563 ± 4,738, *P* < 0.001) concentrations in participants with PE compared to controls. A study that included participants with severe PE reported that the plasma VE concentrations were significantly elevated in women with pregnancy-induced hypertension compared to normotensive pregnant women. Moreover, this study reported increased levels of lipid peroxidation in severely pre-eclamptic women compared to healthy pregnant women (44). In addition, a case–control study reported that the mean plasma VE concentrations were significantly higher in women with PE than in control patients (1.41 ± 0.39 vs. 1.15 ± 0.32 mg/dl, *P* < 0.001) (36). An additional study also reported significantly higher umbilical vein plasma VE concentrations in women with PE than in controls [5.7 (3.6–7.1) vs. 3.6 (3.3–4.5) μmol/L, *P* < 0.001] (45). However, a prospective study reported normal plasma VE concentrations in women with PE and identified a significant positive correlation between gestational age and VE concentrations among both the healthy controls and PE patients (46). A case–control study that included 4,188 pregnant women found higher serum concentrations of VA (*P* < 0.05) in women with PE than in controls (47). Similarly, another study reported that the serum VA concentrations of pregnant women with PE at 12–20 weeks of gestation were higher than those of healthy controls (14). However, a retrospective study found that serum vitamin A and vitamin E levels were negatively correlated with the severity of pre-eclampsia (*P* < 0.001) (48). Therefore, more large-scale studies are needed to verify the relationship between serum concentration of vitamin A, vitamin E, and PE.

Our results identified a discordance between the associations of dietary intake of VA and serum concentrations of VA with PE. Studies have shown that serum VA is associated with dietary VA intake (39). However, in our study, dietary VA intake in the control group was higher than that in the case group, while the serum VA concentration in the control group was lower than that in the case group. The possible reason is that impaired renal function in PE reduces the renal catabolism of the VA carrier retinol-binding protein to transthyretin, thereby increasing circulating VA levels (49). Alternatively, late pregnancy is associated with increased levels of oxidative stress, and VA is a strong non-enzymatic antioxidant in the antioxidant defense system of the human body and has the functions of antioxidation, scavenging free radicals, and anti-apoptosis (47, 50). If the serum VA content is low in pregnant women, it will cause excessive accumulation of free radicals and increase the risk of adverse pregnancy outcomes (48). Therefore, VA might protect against PE *via* such antioxidative effects.

Several lipid-soluble antioxidants, such as VA and VE, may play a mechanistic role in the development of PE, although how these molecules function in this context remains to be determined. Recent studies have shown that altered placental inflammatory status and impaired antioxidative stress pathways may play a role in the pathophysiology of PE (51–53). VA and VE have been implicated as having anti-inflammatory and antioxidative stress properties (54–57), which could reduce the risk of developing PE. A review found that C-reactive protein (CRP) levels decreased after VE supplementation and that acute-phase proteins or proinflammatory cytokines (e.g., CRP and interleukin-6) are markers of inflammation (58). Other studies have suggested that VA and carotenoids can quench singlet oxygen and neutralize sulfhydryl radicals to reduce oxidative stress (59–61). It is, therefore, possible that VA reduces oxidative stress and associated inflammation, thereby reducing the risk of PE (62, 63).

In our study, some limitations should be acknowledged. First, the use of FFQs in dietary surveys may lead to recall bias, limiting the accuracy of our results. Therefore, we conducted a face-to-face survey and used food photographs to help participants to assess their food portions. Second, the information we obtained about dietary intake was based on the participants' recollection of their diet during the first 3 months of the study period, which potentially limited the accuracy. However, our findings can still be used to examine the relationship between dietary VA and VE intake and PE risk, as the time from the onset of PE to delivery is often < 3 months. Third, although we adjusted for possible confounding variables, potentially unknown factors may have influenced the results.

## 5. Conclusion

We found that dietary VA intake was significantly negatively correlated with PE risk, and serum VA and VE concentrations were significantly positively correlated with PE risk among pregnant Chinese women. Further prospective cohort studies and RCTs are warranted to verify these associations.

## Data availability statement

The original contributions presented in the study are included in the article/Supplementary material, further inquiries can be directed to the corresponding author.

## Ethics statement

The studies involving human participants were reviewed and approved by Ethics Committee of Scientific Research and Clinical Trials of the First Affiliated Hospital of Zhengzhou University (No. Scientific research-2016-LW-34). The patients/participants provided their written informed consent to participate in this study.

## Author contributions

YHL, XLZ, and QJL: constructed the study design. YC, DDD, WFD, and WJF: performed the investigation. SPM and XMH: analyzed the data. SPM: drafted the manuscript. YHL, XYW, XYZ, MYG, FFZ, and YCB: reviewed the manuscript. All authors read and approved the final manuscript.
