# Supplementary material for: Dietary intake and serum concentrations of vitamin A and vitamin E and pre-eclampsia risk in Chinese pregnant women: A matched case-control study
Source: Front Nutr. 2023 Mar 30;10:1049055. doi: 10.3389/fnut.2023.1049055 (PMC10101204; doi:10.3389/fnut.2023.1049055)
Supplement: Supplementary file 1 [file Table_1.DOCX]

|  | Q1 | Q2 | Q3 | Q4 | *P*-trend ^b^ |
| --- | --- | --- | --- | --- | --- |
| Dietary vitamin A intake |  |  |  |  |  |
| Cases/controls ^a^ | 141/97 | 101/101 | 73/97 | 67/87 | - |
| Crude OR | 1 | 0.68 (0.46 – 1.00) | 0.56 (0.38 – 0.82) | 0.57 (0.38 – 0.85) | 0.003 |
| Adjusted OR ^c^ | 1 | 0.79 (0.50 – 1.24) | 0.63 (0.40 – 0.99) | 0.64 (0.40 – 1.01) | 0.035 |
| Dietary β-carotene intake |  |  |  |  |  |
| Cases/controls ^a^ | 133/97 | 101/101 | 85/99 | 63/85 | - |
| Crude OR | 1 | 0.73 (0.49 – 1.07) | 0.63 (0.42 – 0.94) | 0.55 (0.36 – 0.83) | 0.004 |
| Adjusted OR ^c^ | 1 | 0.81 (0.52 – 1.27) | 0.61 (0.38 – 0.97) | 0.57 (0.35 – 0.93) | 0.013 |
| Dietary retinol intake |  |  |  |  | - |
| Cases/controls ^a^ | 129/97 | 112/97 | 72/92 | 69/96 |  |
| Crude OR | 1 | 0.85 (0.57 – 1.26) | 0.58 (0.38 – 0.88) | 0.55 (0.37 – 0.83) | 0.002 |
| Adjusted OR ^c^ | 1 | 1.09 (0.68 – 1.75) | 0.63 (0.39 – 1.03) | 0.63 (0.39 – 1.01) | 0.017 |
| Dietary vitamin E intake |  |  |  |  |  |
| Cases/controls ^a^ | 103/97 | 101/100 | 93/94 | 85/91 | - |
| Crude OR | 1 | 0.96 (0.66 – 1.39) | 0.94 (0.65 – 1.37) | 0.89 (0.59 – 1.32) | 0.612 |
| Adjusted OR ^c^ | 1 | 0.90 (0.58 – 1.39) | 0.90 (0.58 – 1.39) | 0.80 (0.51 – 1.26) | 0.379 |

**Supplement Table 1** Odds ratios and 95% confidence intervals of preeclampsia according to quartiles of dietary vitamin A and vitamin E intake among participants without GDM (*n* = 382 pairs).

^a^ Median intake in controls, which were adjusted for daily energy intake.

^b^ Performed by entering the median intake in each quartile as continuous variables in the regression models.

^c^ OR adjusted for age, gestational age, pre-pregnancy BMI, family history of hypertension, education level, parity, physical activity and daily energy intake.
